# Supplementary material for: Functional Analysis of a Novel ABL (Abnormal Browning Related to Light) Gene in Mycelial Brown Film Formation of Lentinula edodes
Source: J Fungi (Basel). 2020 Nov 9;6(4):272. doi: 10.3390/jof6040272 (PMC7712820; doi:10.3390/jof6040272)
Supplement: Supplementary file 1 [file jof-06-00272-s001.tgz › suppplementary materials/Figure S1.pdf]

abl-D	MTLVLSRSGSLSSLSSLSTIATPTTTRTSPPPPHSSLPTGCTSSASASASNHTPRPLQEE
ABL	MTLVLSRSGSLSSISSLSTIATPTTTRTSPPPPHSSLPPGCTSSASASASNHTPRPLQEE
abl-D	KTEPYYADDSYKDPPPPAHVLTGEFGVKIRDFAYEKGGVEVRRVKKYVRPEGQGIGRRRE
ABL	KTEPYYADDSYKDPPPPAHVLASEFGVKIRDFAYEKGGVEVRRVKKYVRPEGQGIGRRRE
abl-D	PVLVQRQPTPPGQRGWMWGVGVGVGGGGGCENAEASTSSGLTRQRVVQDIEDLEFHSQSQ
ABL	PVLVQRQPTPPGQRGWMWGVGVGGGVGGGYENAEASTSSGLTRQRVVQDIEDLEFHSQSQ
abl-D	SQSQSQSQSQPQSLPPFEHISFSESDGEPYIETPAVTPNGSLQWKTTTAEEELTLPSLHN
ABL	SQSQSQSQSQPQSLPPFEHITFSDSDGEPYIETPAVTPNGSLQWKTTTAEEELTLPSLHN
abl-D	LPSSKRAFHPDDDDEDDEQHHTTPPQPPTKKQRVVSPIKRYNLRERRPPATTTTTTLLPP
ABL	LPSSKRAFHPDD-----EQHHTTPPQPPTKKQRVVSPIKRYNLRERRPPTTT----LLPP
abl-D	SRLRNILSVQSSQSQLSTGPKGRRKGKARTTRV
ABL	SRLRNILSVESSQSQLSTGPKGRRKGKARTTRV

Supplementary Figure S1. Amino acid sequence alignment of *abl-D* and *ABL*. Amino acid residues were expressed in one-letter codes and gaps in the aligned sequences were introduced to maximize similarities. Sites where mutation has been identified in *abl-D* are highlighted by colors. Blue, synonymous mutation; Red, missense mutation; yellow, insertion.
